# Forecasting the Incidence of Dementia and Dementia-Related Outpatient Visits With Google Trends: Evidence From Taiwan

**DOI:** 10.2196/jmir.4516

**Published:** 2015-11-19

**Authors:** Ho-Wei Wang, Duan-Rung Chen, Hsiao-Wei Yu, Ya-Mei Chen

**Affiliations:** ^1^ Institute of Health Policy and Management National Taiwan University Taipei Taiwan

**Keywords:** dementia, Alzheimer’s disease, Google Trends, big data, incidence, early detection, self-diagnosis, Internet search, health-seeking behaviors

## Abstract

**Background:**

Google Trends has demonstrated the capability to both monitor and predict epidemic outbreaks. The connection between Internet searches for dementia information and dementia incidence and dementia-related outpatient visits remains unknown.

**Objective:**

This study aimed to determine whether Google Trends could provide insight into trends in dementia incidence and related outpatient visits in Taiwan. We investigated and validated the local search terms that would be the best predictors of new dementia cases and outpatient visits. We further evaluated the nowcasting (ie, forecasting the present) and forecasting effects of Google Trends search trends for new dementia cases and outpatient visits. The long-term goal is to develop a surveillance system to help early detection and interventions for dementia in Taiwan.

**Methods:**

This study collected (1) dementia data from Taiwan’s National Health Insurance Research Database and (2) local Internet search data from Google Trends, both from January 2009 to December 2011. We investigated and validated search terms that would be the best predictors of new dementia cases and outpatient visits. We then evaluated both the nowcasting and the forecasting effects of Google Trends search trends through cross-correlation analysis of the dementia incidence and outpatient visit data with the Google Trends data.

**Results:**

The search term “dementia + Alzheimer’s disease” demonstrated a 3-month lead effect for new dementia cases and a 6-month lead effect for outpatient visits (*r*=.503, *P*=.002; *r*=.431, *P*=.009, respectively). When gender was included in the analysis, the search term “dementia” showed 6-month predictive power for new female dementia cases (*r*=.520, *P*=.001), but only a nowcasting effect for male cases (*r*=.430, *P*=.009). The search term “neurology” demonstrated a 3-month leading effect for new dementia cases (*r*=.433, *P*=.008), for new male dementia cases (*r*=.434, *P*=.008), and for outpatient visits (*r*=.613, *P*<.001).

**Conclusions:**

Google Trends established a plausible relationship between search terms and new dementia cases and dementia-related outpatient visits in Taiwan. This data may allow the health care system in Taiwan to prepare for upcoming outpatient and dementia screening visits. In addition, the validated search term results can be used to provide caregivers with caregiving-related health, skills, and social welfare information by embedding dementia-related search keywords in relevant online articles.

## Introduction

### Background

Dementia is a clinical syndrome caused by neurodegeneration of brain tissue. It encompasses a variety of diseases, including Alzheimer’s disease (AD), vascular dementia, Lewy body dementia, and frontotemporal dementia [1]. It is well known that dementia becomes more prevalent with increasing age, and that this increasing prevalence of dementia is significantly affecting the lives of a large and growing number of older adults around the world. Dementia also generates a substantial effect on public health, the social care system, and societal costs [2].

Worldwide, the number of people over the age of 60 is projected to increase to 1.25 billion by 2050, and will account for 22% of the total world population [3]. According to the World Alzheimer Report released by Alzheimer’s Disease International (ADI), nearly 36 million people worldwide are believed to have been living with AD or other dementias in 2013; that number is expected to reach 66 million by 2030 and to increase to more than 115 million by 2050 [4]. ADI reported that overall payments for people aged 65 years and older with dementia—including health care, long-term care, and hospice services—are expected to reach US $203 billion in 2013, increasing to US $1.2 trillion by 2050 [1]. Additionally, the Aging, Demographics, and Memory Study (ADAMS), a nationally representative study taking place in the United States, recently reported that the yearly societal costs in the United States attributable to dementia amounted to US $41,689 per person, with 68% of the total costs stemming from direct costs for health and social care, and the remainder due to the cost of informal care [5].

Informal caregivers have recently become an important target group for long-term care policy making. The Organisation for Economic Co-operation and Development (OECD) and other researchers have suggested that informal caregivers need to learn and obtain more skills for care and more knowledge to avoid the depression and isolation which could potentially lead to more medical utilization [6-8]. However, a challenge that has been raised in much of the literature regarding caregivers has been identifying this group of informal caregivers and determining a way to reach out to them [9,10].

Although there is no known cure for dementia, some pharmaceutical drugs, treatments, and activity interventions may potentially help to improve or maintain the symptoms [11-13]. Thus, it has been suggested that these treatments and interventions be delivered at an earlier stage of dementia, rather than at a later stage, to achieve maximum benefits, including reduced direct medical costs [12,13], delayed institutionalization [3], and maintenance of lower symptom severity levels for longer periods [14,15]. However, current diagnoses tend to be made at a relatively later stage in the course of the disease, partly due to the lack of awareness of symptoms associated with dementia, as well as to the stigma and denial of its existence [16,17]. Under such serious financial burdens on society, all governments and the World Health Organization are set to establish dementia as a public health priority in order to reach out to individuals with early-stage dementia and their caregivers through creative strategies [18].

One of the main challenges of dementia is the lack of appropriate tools and channels for helping individuals identify themselves as having symptoms of dementia, as well as for their family members to quickly examine those they care for. The stigma associated with dementia increases this challenge [17]. A dementia diagnosis is generally perceived negatively by both family members and patients. Stigma and stereotypes are significant obstacles to well-being and quality of life [18-21]. The high rates of Internet searches for health information have suggested that the Internet may be an important resource for patients and family members, who may try to access information for self-diagnosis first, rather than visit their physicians for a more formal diagnosis [22]. Thus, the possibility of reaching out to people with dementia and their family members through the Internet merits further study.

In recent years, the Internet has become a popular medium for people searching for health-related knowledge and information for self-diagnosis [23]. A recent online health survey conducted in the United States reported that in 2012, 35% of US adults looked online to figure out medical conditions, and 72% of Internet users looked online for health information [22]. Of these online health seekers, 77% began at a search engine such as Google or Yahoo. About half of all online health searches are reportedly made on behalf of someone else [22]. These increasing numbers of online searches through search engines creates trend data which can be analyzed in real time [24].

### Previous Research

The use of aggregated search queries has considerable potential for syndromic surveillance, as proven by comparing the evidence found in the numbers of clicks on a keyword-triggered link in Google with the epidemiological data from the 2004-2005 flu season in Canada [25]. Since 2004, Google has provided two services for trend analysis: Google Flu Trends (GFT) and Google Trends (GT). In a landmark study published in the journal Nature, GFT has been identified as a powerful tool used in influenza surveillance in the United States, identifying influenza epidemics up to 7-10 days before detection by the Centers for Disease Control and Prevention's (CDC’s) influenza surveillance network [26]. During periods of infectious disease prevalence, GFT has also been able to predict emergency department visit volumes [27,28].

However, GFT failed to correctly estimate the scale of the 2009 H1N1 pandemic in the United States. Several mistakes that led to GFT’s overestimation of H1N1 incidence were caused by the limited transparency of Google’s treatment of data and its dynamic algorithm, due to Google’s business considerations [29,30]. To overcome this, GFT recently used aggregated Google search data in a model created in collaboration with the CDC to estimate influenza activity in the United States, and the results are available in the CDC’s weekly US Influenza Surveillance Report [31].

Following the same logic, GT can be extended to any area for researchers to graph the frequency of searches for a specific term or phrase [24]. These graphs are normalized on a relative basis and can also be restricted to specific time intervals or geographic regions [32]. GT data is available in the United States for city, country, or subnational areas; but for other countries worldwide, including Taiwan, GT data is mostly available on a national basis [24,33]. By tracking health-seeking behavior, GT has predictive capability to monitor the epidemic curve of food-borne illnesses, such as peanut butter-associated outbreaks of *Salmonella enterica* serotype Typhimurium [34], as well as the incidence of human immunodeficiency virus [19,35].

This tool has more recently been extended to study the relationships among macroeconomic conditions and mental illness. A recent study reported that a 5% rise in the unemployment rate is followed in the next 12 months by an approximate 14% increase in searches around problem drinking [36]. Another study showed that searches for key terms such as “divorce,” “asthma,” and “social welfare” led the suicide death data for 2 months [23]. Additionally, a previous study demonstrated how restaurant table availability has the potential to monitor the incidence of influenza-like illness [37]. Nowadays, this type of research has become a new discipline collectively termed *infodemiology* [25,38,39]. Based on GT’s algorithm, mechanism, and research findings, it may be expected that what the general public searches for today will have predictive power for what will occur in the near future.

### Research Goals

To the best of our knowledge, most previous studies of GT data have been in the field of infectious diseases, and quite a few have been in the field of noncommunicable and chronic disease. Additionally, most of the previous studies took place in the United States. By contrast, such studies in Asia, and in Taiwan in particular, are more rare.

The first objective of this study was to investigate and validate the search terms that could be the best predictors of new dementia cases and outpatient visits. The second objective was to further evaluate the lead pattern of GT search trends related to new dementia cases and outpatient visits. The long-term goal is to contribute to developing a surveillance system for the early detection of dementia in Taiwan.

## Methods

### Data Sources

#### Overview

Data on dementia in Taiwan from January 1, 2009 to December 31, 2011, was obtained from the Longitudinal Health Insurance Database 2010 (LHID 2010), a subset of the National Health Insurance Research Database (NHIRD). These data were collected and maintained by the National Health Insurance Institutes [40]. The NHIRD has comprehensive claim data on outpatient and inpatient services from approximately 27.38 million individuals enrolled in Taiwan’s National Health Insurance program. The LHID 2010 contains the entire original claim data of 1 million beneficiaries enrolled in the year 2010 and randomly sampled from the year 2010 Registry for Beneficiaries (ID) of the NHIRD.

#### New Dementia Cases

Monthly figures of new dementia cases were obtained from a data file named Ambulatory Care Expenditures by Visits (CD), which is a subset database of the LHID 2010. Patients with a dementia diagnosis were identified using the International Classification of Disease, Ninth Revision, Clinical Modification (ICD-9-CM), including the diagnosis codes 290.x, 294.x, and 331.x. Patients aged 65 years or over who had received a dementia diagnosis between the years of 2009 and 2011 were included in this study. To avoid the miscounting of new case numbers, the data were reviewed and cases in which individuals had been diagnosed as dementia patients from 2005 through 2008 were excluded from the study. A total of 5383 new dementia cases were identified, with 2442 (45.37%) male cases and 2941 (54.63%) female cases.

#### Dementia-Related Outpatient Visits

Dementia-related outpatient visits include both regular dementia-related clinical visits and clinical visits with new dementia diagnoses. Total visits from 2009 to 2011 were obtained from the CD data file. The numbers of monthly outpatient visits related to dementia and attributed to patients aged 65 years or older were calculated using the same ICD-9-CM criteria described above. A total of 113,710 outpatient visits were identified between the years of 2009 and 2011.

#### Google Trends Search Terms and Trends

GT was provided by Google Inc starting in 2004 [24]. Search trends in GT over time are represented as a scaled number (0-100) which is normalized to the time of maximal value for those particular search terms, thus allowing for comparisons between the search terms.

To capture a broad sense of dementia-related conditions and services, 23 search terms were catalogued through GT. The relevant terms were derived from GT’s explore function and included disease names, symptoms, care services, and hospital divisions. For example, disease names catalogued as search terms included "dementia," "Alzheimer’s disease," and "Parkinson’s disease" (see Table 1). GT only reports results above a certain threshold. When GT could not report the search volume for a term, the message displayed was “Not enough search volume to show graphs.” Those search terms with insufficient search volume compared to GT’s threshold were excluded. Data on the remaining three search terms from January 2009 to December 2011 (36 months) were accessed and downloaded from GT on December 28, 2013. Table 1 shows the list of dementia-related categories and search terms; Multimedia Appendix 1 shows these terms in Chinese, along with their English equivalents.

**Table 1:** List of dementia-related search terms.

Category	Search terms^a^
Disease terms	Dementia; Alzheimer's disease; Parkinson's disease; senile dementia of the Alzheimer type; vascular dementia; amnesia; anxiety disorder; depression
Symptom terms	Dementia; senile dementia; geriatric dementia; sunset phenomenon; forgetful; memory; insomnia
Care terms	Dementia care; dementia respite; Taiwan Alzheimer dementia association; school of wisdom; dementia care; caregiver; long-term care
Division term	Neurology

^a^Search terms are shown in their English equivalents; see Multimedia Appendix 1 for the terms in Chinese.

### Statistical Analyses

#### Overview

Pearson correlation coefficient analysis is widely used in GT research [41-43]. To access the strength of the linear relationship between search trends and new dementia cases and outpatient visits, Pearson correlation coefficient analysis with 95% CI was performed for all the data. The two steps to the analysis are described below. All statistical analyses were conducted using IBM SPSS Statistics for Windows, version 20.0 (IBM Corp, Armonk, NY); a two-tailed *P* value of less than .05 was required for statistical significance in all analyses conducted. This study was approved by the National Taiwan University Hospital (NTUH) Research Ethics Committee (REC).

#### Step I: Temporal Trends

The temporal relationship between GT search trends and overall new dementia cases and outpatient visits was analyzed first. GT’s lead pattern analysis was then analyzed by 1-month, 3-month, 6-month, 9-month, and 12-month intervals. For example, a 1-month lead evaluates the correlation of GT search trends from January 2009 with new dementia cases and dementia-related outpatient visits in February 2009.

#### Step II: Correlation Analysis

To account for differences in the GT lead patterns influenced by gender, a correlation between GT search trends and new dementia cases and outpatient visits was first analyzed by gender, after which the analysis followed the lead pattern analysis described in Step I.

## Results

### Temporal Trends

**Figure 1:**
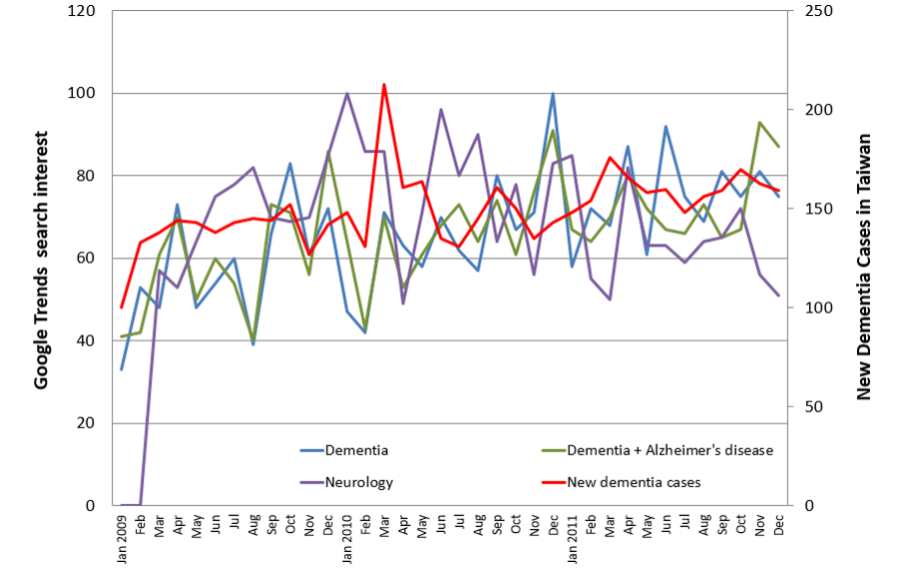
Time series for the monthly counts of new dementia cases in Taiwan between January 2009 and December 2011 plotted with Google Trends search terms.

**Figure 2:**
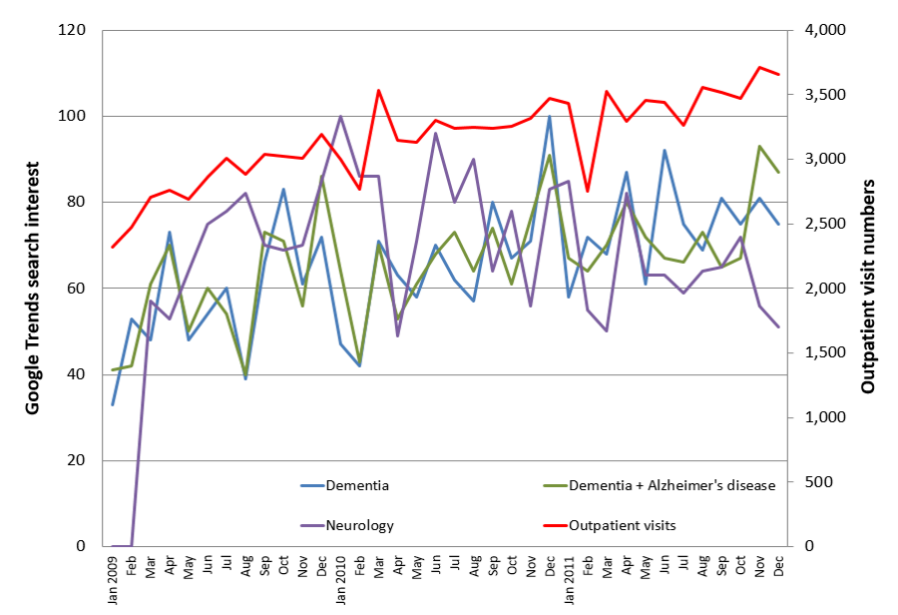
Time series for the monthly counts of outpatient visits between January 2009 and December 2011 plotted with Google Trends search terms.

**Figure 3:**
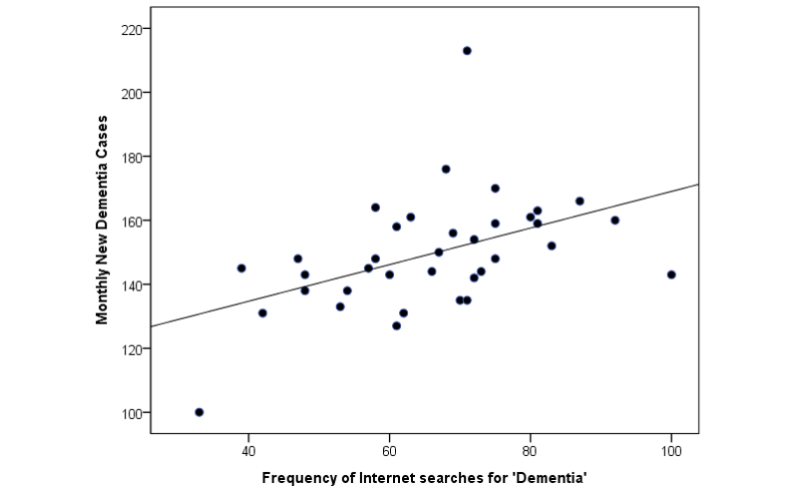
New monthly dementia cases and search trends of "dementia" in Taiwan from 2009 to 2011.

**Figure 4:**
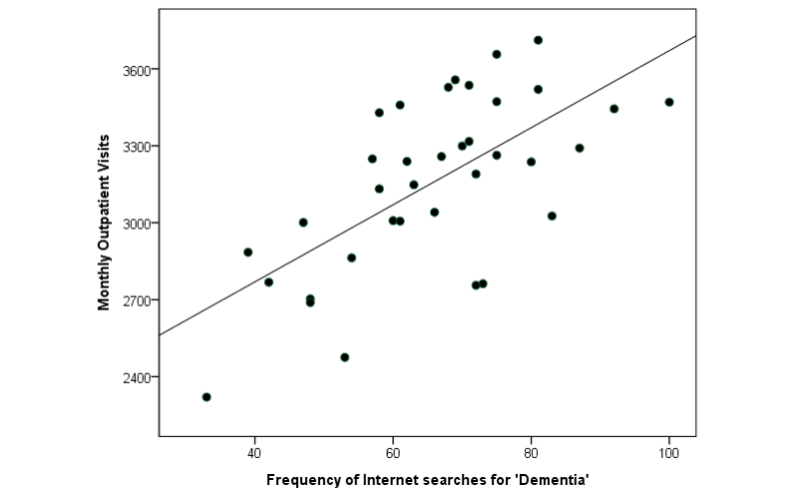
Monthly outpatient visits and search trends of "dementia" in Taiwan from 2009 to 2011.

Only three key terms—“dementia,” “Alzheimer’s disease,” and “neurology”—came above the GT threshold and were recorded in GT. The other 20 terms either did not generate sufficient search volume, and thus were excluded from GT, or were not relevant to dementia. GT reported that searches for “dementia” and “dementia + Alzheimer’s disease” steadily increased throughout the study period. Figures 1 and 2 illustrate the temporal relationship between search term trends and the number of new dementia cases (Figure 1) and outpatient visits (Figure 2). Scatterplots were constructed to compare new dementia cases and outpatient visits with data gathered from GT (see Figures 3 and 4).

### Correlation Analysis

From the comparisons of new dementia cases and outpatient visits with search data gathered from GT, Pearson correlation coefficient analysis was conducted.

#### Nowcasting Effect

*Nowcasting*, also known as contemporaneous forecasting, is a term used to describe predicting the present. Nowcasting has become widely popular in economics because of the significant time lag in statistical releases [44]. The results of the cross-correlation analysis of new dementia cases and GT data suggested that the search term “dementia” moderately coincided with new dementia cases (*r*=.469, *P*=.004). For outpatient visits, the search terms “dementia” and “dementia + Alzheimer’s disease” both indicated strong correlation (*r*=.658, *P*=.001; *r*=.727, *P*=.001, respectively; see Table 2) with outpatient visit numbers. When gender differences were considered, “dementia,” but not “dementia + Alzheimer’s disease,” moderately coincided with new male dementia cases (*r*=.430, *P*=.009). These results demonstrate the possibility of monitoring the scale and the temporal and spatial patterns of online search behaviors that may plausibly be associated with future clinical diagnoses of dementia.

**Table 2:** Pearson cross-correlation analysis of new dementia cases, outpatient visits, and search trends, 2009-2011.

Search term and variable		Pearson cross-correlation coefficient^a^ (*P*)					
		Search preceded outpatient visit or new diagnosis by...					
		No lag	1 month	3 months	6 months	9 months	12 months
**Dementia**							
	New dementia cases	.469 (.004)	.118 (.49)	.393 (.02)	.365 (.03)	.130 (.45)	.068 (.70)
	New male dementia cases	.430 (.009)	.079 (.65)	.093 (.59)	.015 (.93)	.064 (.71)	-.059 (.73)
	New female dementia cases	.297 (.08)	.100 (.56)	.489 (.002)	.520 (.001)	.131 (.45)	.152 (.38)
	Outpatient visits	.658 (.001)	.542 (.001)	.554 (.001)	.349 (.04)	.331 (.048)	.365 (.03)
**Dementia + Alzheimer's disease**							
	New dementia cases	.385 (.02)	-.005 (.98)	.503 (.002)	.348 (.04)	.145 (.40)	.111 (.52)
	New male dementia cases	.358 (.03)	-.065 (.70)	.257 (.13)	.109 (.53)	-.134 (.44)	-.102 (.55)
	New female dementia cases	.239 (.16)	.052 (.76)	.502 (.002)	.410 (.01)	.332 (.048)	.255 (.13)
	Outpatient visits	.727 (.001)	.394 (.02)	.514 (.001)	.431 (.009)	.228 (.18)	.317 (.06)
**Neurology**							
	New dementia cases	.263 (.12)	.304 (.07)	.433 (.008)	.112 (.52)	.227 (.18)	.154 (.37)
	New male dementia cases	.340 (.04)	.371 (.03)	.434 (.008)	.081 (.64)	.121 (.48)	.216 (.21)
	New female dementia cases	.077 (.66)	.108 (.53)	.240 (.16)	.090 (.60)	.223 (.19)	.030 (.86)
	Outpatient visits	.393 (.02)	.421 (.01)	.613 (<.001)	.356 (.03)	.245 (.15)	.267 (.12)

^a^Values represent cross-correlation coefficients where .8 was operationally defined as an excellent correlation, .6-.8 indicated good correlation, .4-.6 indicated a moderate correlation, and ≤.4 indicated a poor correlation.

#### Forecasting Effect

Separate from nowcasting, the forecasting effect aims to catch how far in advance GT could help predict the future. The results of applying GT lead patterns to search terms suggested that the search terms “dementia + Alzheimer’s disease” and “neurology” led the overall new dementia cases reported in the LHID 2010 by 3 months (*r*=.503, *P*=.002; *r*=.433, *P*=.008, respectively). For outpatient visits, two search terms—"dementia” and “neurology”—demonstrated a 3-month leading effect, and the search term “dementia + Alzheimer’s disease” demonstrated a 6-month leading effect (see Table 2). Moreover, there was an interesting finding regarding gender differences. The search term “dementia” showed 6-month predictive power for the new female dementia cases (*r*=.52, *P*=.001), but not for the male cases. However, the search term “neurology” showed a 3-month predictive power for the new male dementia cases (*r*=.434, *P*=.008), but not for the female cases. This implies different Internet search behaviors, whether performed by individuals or caregivers, for male and female dementia patients.

## Discussion

### Principal Findings

This study contributes to an understanding of how GT search trends are related to dementia cases and outpatient visits by analyzing data gathered between the years of 2009 and 2011 in Taiwan. Furthermore, this study’s results demonstrate the possibility of monitoring the scale and the temporal and spatial patterns of online search behaviors that may plausibly be associated with future clinical diagnoses of dementia.

Regarding nowcasting capability, findings showed that GT data temporally coincided with the number of new dementia cases and outpatient visits. This finding is consistent with prior research showing that GT provides near-real-time surveillance data up to 7-10 days in advance of CDC’s influenza surveillance network [26]. As for long-term forecasting capability, three search terms—“dementia,” “dementia + Alzheimer’s disease,” and “neurology”—had leading effects on reported new dementia cases and outpatient visits. This finding also confirms previous studies’ results, which suggested that a set of suicide-related search terms, whose trends either temporally coincided with or preceded trends in suicide data, were associated with suicide death [23].

Another important finding in this study is that the search term “dementia” yielded more sensitivity in prediction power among women (with a 6-month leading effect) than among men. This finding may be reasonably explained by a recent study which suggested that women would be more likely than men to report subjective memory complaints (SMCs), which is one of the earliest symptoms observed in the prodromal phase of dementia, whereas men would be more likely to report difficulties or restrictions in instrumental activities of daily living (IADLs), which may become apparent at a later stage of the dementia diagnosis [45].

According to statistics from the Ministry of Health and Welfare Taiwan, in 2012, female patients generated more than 1.55 times the number of mental disorder clinical visits than male patients [46]. This indicates that female patients and their families may possibly take the step of going to see a doctor for complaints earlier than male patients do. In addition, men usually are not expected to fulfill IADLs such as housekeeping, cooking, and washing clothes, so once restrictions in instrumental activities are noted and reported, the dementia will likely be at a later stage [47]. Therefore, we interpreted the results of the search term “dementia” to indicate that families of women with dementia would search online for dementia-related information or knowledge at an earlier stage than would families of men with dementia. Conversely, owing to the stigma and stereotypes of dementia [18], men tended to search for “neurology” instead of “psychiatry” in Taiwan [46]. This may reasonably explain why the search term “neurology” yielded more sensitivity in prediction power among men (with a 3-month leading effect) than among women.

In Taiwan, dementia prevalence at ages 65-69 was 3.4% in 2013 [48]. In Taiwan in 2015, according to a survey by ClickForce [49], just 5.2% of adults aged 55 and over had at least one experience of searching on the Internet. Therefore, in this study we assumed that most searches were initiated by family members, whether before or after patient diagnosis. In addition, we anticipated that searches originating from dementia patients themselves would reflect concurrently in a *no lag* period after their outpatient visits, rather than precede a visit or a new diagnosis.

Most people in Taiwan have the basic knowledge to understand disease-related divisions in hospitals, such as neurology, and this is believed to be related to the increasing tendency for patients and family members to use Internet searches for health information prior to seeing a doctor. Such search trends can now be applied as leading indicators which are capable of predicting disease incidence and related increases in outpatient visits earlier than conventional surveillance [22,24,32].

In comparison with conventional surveillance methods, GT has several advantages. First, GT is a free and easily accessible search tool. Second, GT is now available for more than 70 countries worldwide, and thus allows for tracking of searches in different languages and regions. Last, GT is updated weekly, which permits more frequent monitoring of symptoms and could facilitate early detection of dementia. In this regard, GT can be used to notify health policy makers and hospital managers to get ready for upcoming outpatient visits, where screening work is expected to achieve early detection and treatment and thus save more medical and care costs [50,51].

Additionally, from a public health point of view, health policy makers and health care professionals may consider using key search terms to inversely feed dementia-related information, such as health care resources, care skills, and social welfare information, to those formal and informal caregivers who are difficult to identify in the dementia care system. It is possible to embed dementia-related keywords in online articles, where they can be easily found and recorded by Googlebot—Google’s Web-crawling bot that discovers new and updated pages to be added to the Google index—and thus delivered to users who search for these keywords on the Internet [52]. These users are likely to be the people conducting dementia-related searches on behalf of someone they care for. Using these inverse search strategies to deliver information may improve various aspects of caregivers’ well-being, such as increasing confidence and reducing depression, as well as lowering the potential utilization of medical and health care services by caregivers of persons with dementia [6-8].

### Further Research and Limitations

Our research suggests the need for the following further research. First, further qualitative research is needed to validate the results of this study. In particular, an examination of the Internet search behaviors of caregivers for people diagnosed with dementia is necessary. Also, the gender difference reported in the argument on early report of SMC by female patients needs validation [45]. Second, additional research at different geographic scales would be worthwhile, in places where GT offers complete subnational data points. Third, aside from GT, Google AdWords and Baidu trends also have the potential to play similar roles in the surveillance of dementia, and research on these tools merit further study. Lastly, more dementia-related keywords and a longer period of data points merit further study to validate GT’s lead pattern. This study contributes to the literature by expanding the GT research field to the disease of dementia. To the best of our knowledge, no existing empirical research addresses the relationship between GT and dementia or ways of estimating potential medical needs for people with Alzheimer's disease.

Several limitations of this study should be noted, mostly related to the GT algorithm. First, owing to insufficient and incomplete data sources, only 3 years of GT data were included in this study. Future research with longer periods of data points is recommended to validate the lead patterns. Second, there was difficulty in identifying search trends that were generated by true cases. In particular, GT tends to be influenced by media exposure of specific diseases (eg, drug advertisements), which drives more nonpatients to search related terms and thus increases the search volumes [53]. Lastly, the calculation of GT depends on Google’s assumptions and normalization, which are not clearly reported.

### Conclusions

In conclusion, GT serves as an easily accessible, real-time surveillance tool. Despite the limitations, this study highlights GT as a useful tool for establishing a plausible relationship between search terms and new dementia cases and dementia-related outpatient visits. This tool can lead to better management of medical resources and budgets in Taiwan’s health and social care system. It is possible that GT could be further developed as a surveillance platform to help lower the individual and social costs of this troublesome disease associated with aging populations.

## Appendix

Multimedia Appendix 1 List of Chinese dementia-related search terms and their English equivalents.

## Abbreviations

AD: Alzheimer’s disease
ADAMS: Aging, Demographics, and Memory Study
ADI: Alzheimer’s Disease International
CD: Ambulatory Care Expenditures by Visits
CDC: Centers for Disease Control and Prevention
GFT: Google Flu Trends
GT: Google Trends
IADLs: instrumental activities of daily living
ICD-9-CM: International Classification of Disease, Ninth Revision, Clinical Modification
ID: Registry for Beneficiaries
LHID 2010: Longitudinal Health Insurance Database 2010
NHIRD: National Health Insurance Research Database
NTUH: National Taiwan University Hospital
REC: Research Ethics Committee
SMC: subjective memory complaint
