# Supplementary material for: Forecasting the Incidence of Dementia and Dementia-Related Outpatient Visits With Google Trends: Evidence From Taiwan
Source: J Med Internet Res. 2015 Nov 19;17(11):e264. doi: 10.2196/jmir.4516 (PMC4704919; doi:10.2196/jmir.4516)
Supplement: Multimedia Appendix 1 [file jmir_v17i11e264_app1.pdf]

Multimedia Appendix 1. List of Chinese dementia-related search terms and their English equivalents.

English equivalent	Original Chinese search terms
Disease terms	
Dementia	失智症
Alzheimer’s disease	阿茲海默
Parkinson’s disease	帕金森
Senile dementia of the Alzheimer type	腦退化症
Vascular dementia	血管失智
Amnesia	健忘症
Anxiety disorder	焦慮症
Depression	憂鬱症
Symptom terms	
Dementia	失智
Senile dementia	老人癡呆
Geriatric dementia	老年癡呆
Sunset phenomenon	黃昏症候群
Memory	記憶力
Forgetful	健忘
Insomnia	失眠
Care terms	
Dementia care	失智照護
Dementia respite	失智喘息
Taiwan Alzheimer dementia association	失智協會
School of wisdom	瑞智學堂
Dementia care	失智照護
Caregiver	看護
Long-term care	長期看護
Division term	
Neurology	神經內科
